# First person – Vinoth S

**DOI:** 10.1242/bio.062137

**Published:** 2025-08-11

## Abstract

First Person is a series of interviews with the first authors of a selection of papers published in Biology Open, helping researchers promote themselves alongside their papers. Vinoth S is first author on ‘ A pro-angiogenic and hypoxic zebrafish model as a novel platform for anti-angiogenic drug testing’, published in BiO. Vinoth conducted the research described in this article while a PhD research scholar in Dr S. Kirankumar's lab at SRM Institute of Science and Technology, Kattankulathur, Chennai, India. He is now a project scientist-I in the lab of Dr Rajeeb K. Swain at the Institute of Life Sciences, Bhubaneswar, India, investigating biotechnology and specialising in disease modelling using zebrafish and gene-editing technologies to support therapeutic development and drug testing.


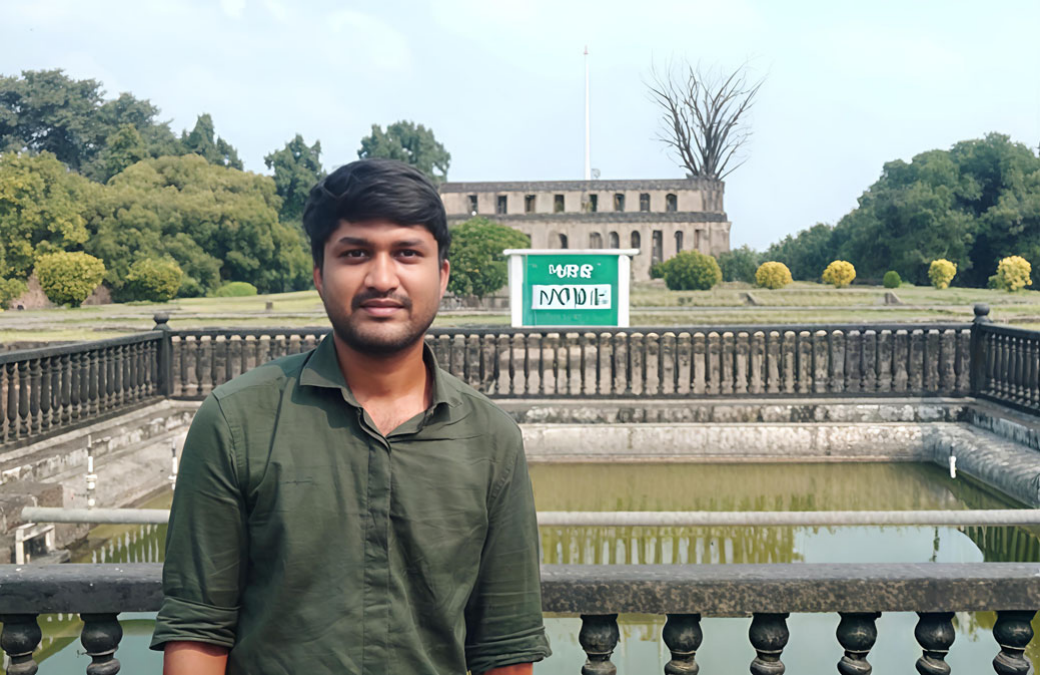


**Vinoth S**

**Describe your scientific journey and your current research focus**

My scientific journey began with a bachelor's project focused on bioremediation using basic microbiological techniques. This early exposure sparked my interest in applying science to real-world problems. During my master's program, I had the opportunity to work at the National Centre for Aquatic Animal Health, Cochin University of Science and Technology, India, where I focused on gene silencing studies. Building on that experience, I continued as a project assistant in the same lab on various aquaculture-based research projects, which helped strengthen my foundation in both molecular biology and aquatic research. I then pursued my PhD at SRM Institute of Science and Technology, where I worked on developing a zebrafish pseudohypoxia model. Currently, I am working as a project scientist-I at the Institute of Life Sciences (ILS), focusing on the development of Type I and Type II diabetes models using zebrafish.

**Who or what inspired you to become a scientist?**

To be honest, it wasn't a person or event that inspired me, it was my own interest in getting educated. My decision to enter the field of biological sciences was shaped by the opportunities available at the time. Over time, I've grown to enjoy what I do and feel motivated to contribute meaningfully with the chances I get.

**How would you explain the main finding of your paper?**

Cancer treatments often fail because some tumours become resistant to drugs, especially in hypoxic environments. Our research used zebrafish, a small freshwater fish often used in biomedical research, to study how hypoxia affects the response to anti-cancer drugs. We created pseudo-hypoxia conditions in zebrafish using chemical and genetic approaches and tested a drug called sorafenib, which is designed to block the growth of blood vessels that feed tumours. We discovered that hypoxia activated certain genes and signals that protected the blood vessels to keep growing, even in the presence of the drug. This shows that hypoxic regions in tumours can make cancer cells more resistant to treatment. Our zebrafish model highlights the role of hypoxia in drug resistance and could help improve future cancer drug testing.

**What are the potential implications of this finding for your field of research?**

In our study, we hypothesised that the efficacy of antiangiogenic compounds might differ in hypoxic tissue environments compared to normal tissues. Traditionally, drug testing has been conducted using normal zebrafish larvae, which do not exhibit the counteracting responses observed in hypoxic tumours. To test this, we developed a zebrafish hypoxia model and found that anti-cancer drugs like sorafenib further elevated hypoxia-related signalling, rendering the drug less effective and potentially exacerbating the disease condition. Therefore, our zebrafish hypoxia model offers a biologically meaningful platform for antiangiogenic drug testing and could contribute to the development of more effective cancer therapeutics. This study represents a step forward from using normal zebrafish larvae by introducing a hypoxia-based zebrafish model that more closely mimics tumour hypoxia conditions for drug screening.

**Figure:**
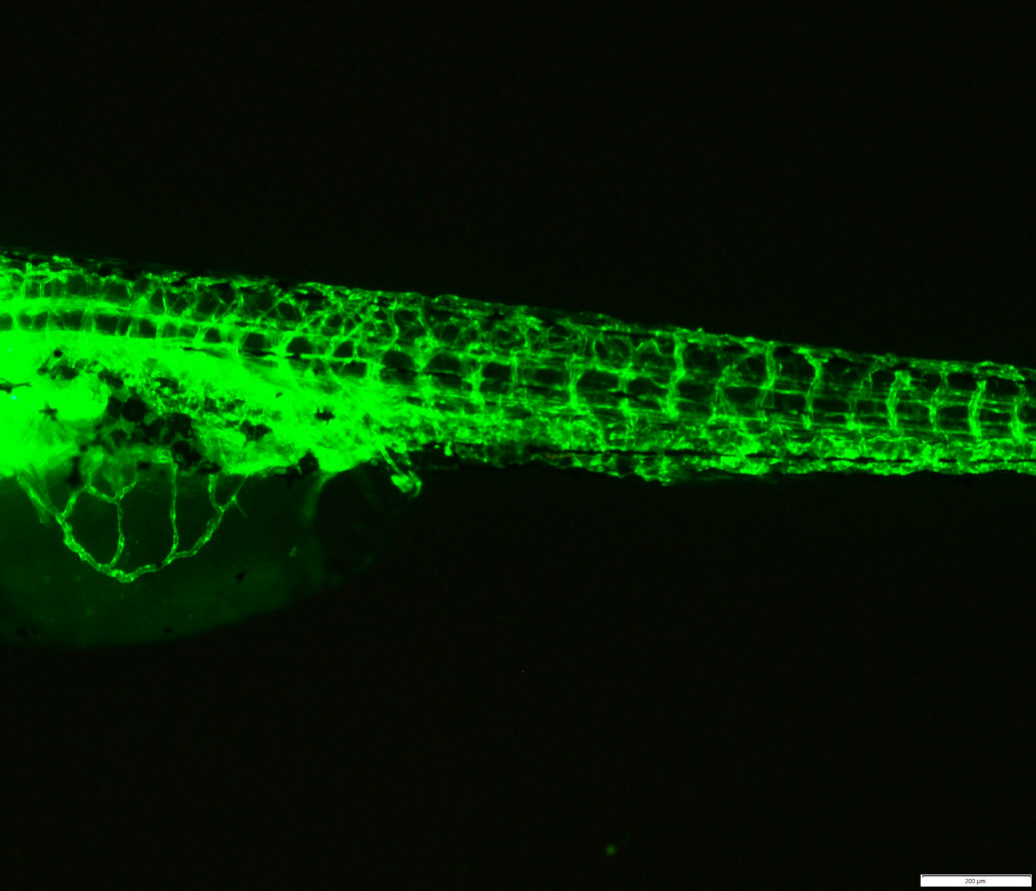
Inverted phase-contrast fluorescent image showing blood vessel network in zebrafish larvae.

**Which part of this research project was the most rewarding?**

One of the most rewarding moments was identifying how our model overcomes the effects of the drug – an exciting breakthrough. Also, this work gave me the opportunity to present at the Indian Zebrafish Investigators Meeting (iZIM 2024), where I saw a similar level of enthusiasm from many leading scientists. I received the same encouraging feedback during my PhD defence and from reviewers throughout the manuscript review process. We hope this work contributes to reshaping how anti-angiogenic drug testing is approached using the zebrafish model within the research community.

One of the most fulfilling aspects is being able to guide fellow researchers and mentor budding scientists

**What do you enjoy most about being an early-career researcher?**

What I enjoy most about being an early-career researcher is the sense of confidence and capability I've developed over time. With a solid foundation of skills, I no longer struggle to learn new techniques – instead, I can approach research problems with clarity and work more efficiently. I also value the continuous process of upgrading my knowledge and refining my approach to scientific questions. One of the most fulfilling aspects is being able to guide fellow researchers and mentor budding scientists, which not only reinforces my own understanding but also makes me feel more capable of contributing meaningfully to the scientific community.

**What piece of advice would you give to the next generation of researchers?**

It's natural to hear mixed things about pursuing a PhD or a career in science, but don't let that discourage you. The journey can be manageable if you stay sincere, work diligently, and avoid overcomplicating things. And remember, there are always people around who are willing to guide and support you along the way. Trust the process and grow at your own pace.

**What's next for you?**

After one more focused postdoctoral research experience, I aim to transition into an industrial setting where I can apply the zebrafish model as a robust platform for drug screening.

**How do you like to be remembered at the end of the day?**

At the end of the day, I'd like to be remembered as someone who showed up with sincerity, stayed committed to the work, and supported others without hesitation. Whether it's solving a problem, sharing knowledge, or simply being a reliable team member, I hope people feel that I contributed with integrity and helped make things a little better – both scientifically and personally.
